# Preferences of physicians for treatment-related toxicity vs. recurrence in melanoma (GERMELATOX-A): the doctors’ perspective

**DOI:** 10.1007/s00432-024-05713-6

**Published:** 2024-05-14

**Authors:** Katharina C. Kähler, Ralf Gutzmer, Yenny Angela, Elisabeth Livingstone, Georg Lodde, Frank Meiss, David A. Rafei-Shamsabadi, Sera S. Weyer-Fahlbusch, Dorothée Nashan, Carmen Loquai, Jessica C. Hassel, Michael M. M. Sachse, Lara V. Maul, Lucie Heinzerling, Markus V. Heppt, Chiara Colapietro, Judith Rusch, Christine Blome

**Affiliations:** 1 Department of Dermatology, University Hospital Schleswig-Holstein (UKSH), Campus Kiel, Kiel, Germany; 2 Department of Dermatology, Johannes Wesling Medical Center Minden, Ruhr University Bochum Medical School, Bochum, Germany; 3 Department of Dermatology, University Hospital Essen, Essen, Germany; 4 Department of Dermatology, Faculty of Medicine, Medical Center-University of Freiburg, University of Freiburg, Freiburg, Germany; 5 Department of Dermatology, Dortmund, Germany; 6 Department of Dermatology, Klinikum Bremen-Ost, Gesundheitnord gGmbH, Bremen, Germany; 7 Medical Faculty Heidelberg, Department of Dermatology and National Center for Tumor Diseases (NCT), Heidelberg University, NCT Heidelberg, a partnership between DKFZ and University Hospital Heidelberg, Heidelberg, Germany; 8 Department of Dermatology, Bremerhaven, Germany; 9 Department of Dermatology, University Hospital Basel, Basel, Switzerland; 10 Department of Dermatology, University Hospital Zurich, Zurich, Switzerland; 11 Department of Dermatology and Allergy, University Hospital, LMU Munich, Munich, Germany; 12 Department of Dermatology, Uniklinikum Erlangen, Friedrich-Alexander University (FAU) Erlangen-Nürnberg, University Hospital Erlangen, Erlangen, Germany; 13 Institute for Health Services Research in Dermatology and Nursing (IVDP), University Medical Center Hamburg-Eppendorf, Hamburg, Germany; 14 Comprehensive Cancer Center Erlangen-European Metropolitan Area of Nuremberg (CCC ER-EMN), Erlangen, Germany

**Keywords:** Physician preferences, Melanoma, Treatment toxicity, Adjuvant treatment

## Abstract

**Introduction:**

Adjuvant treatment with immune checkpoint inhibitors, such as PD1-antibodies (ICI) ± CTLA4-antibodies (cICI) or targeted therapy with BRAF/MEK inhibitors (TT), has shown a significant improvement in disease-free survival (DFS) for high-risk melanoma patients. However, due to specific side effects, the choice of treatment is often influenced by the risk of toxicity. Therefore, the role of physicians in treatment decisions of patients is crucial. This study investigated for the first time in a multicenter setting the attitudes and preferences of dermatooncologists in Germany and Switzerland regarding adjuvant treatment with (c)ICI and TT.

**Methods:**

In the GERMELATOX-A study, 108 physicians (median age: 32 yrs, 67.6% female) from 11 skin cancer centers were surveyed to rate typical side effect scenarios of (c)ICI and TT treatments and then compared to patients’ ratings evaluated in a previous analysis from the same centers. The scenarios described mild-to-moderate or severe toxicity and included melanoma relapse leading to death. The physicians were asked about the level of side effects they would tolerate in exchange for a reduction in melanoma relapse and an increase in survival at 5 years.

**Results:**

The preferences of physicians and patients revealed significant differences regarding adjuvant melanoma treatment with (c)ICI and TT (*p* < 0.05). Compared to patients, physicians tend to value a melanoma relapse less severe, according to a visual analog scale. They were also less threatened by all scenarios of side effects during adjuvant treatment with (c)ICI or TT, compared to patients. Physicians required lower risk reductions for disease-free survival (DFS) and overall survival (OS) for both ICI and TT and their drug-related side effects to accept these treatments. In case of severe side effects, physicians required similar 5-year DFS rates for ICI and TT (60–65%), while patients needed a 15% improvement of 5-year DFS for ICI compared to TT (80%/65%). For survival, physicians expected an OS improvement of + 10% for all three treatment modalities, whereas patients required a higher increase: + 18–22% for ICI and + 15% for TT.

**Conclusion:**

Our study highlights the importance of understanding the patient’s perspective and a potential difference to the doctor’s view when making decisions about adjuvant melanoma treatment with (c)ICI and TT, especially as these treatments are increasingly being implemented in earlier stages.

## Introduction

Over the years, significant advancements have been made in the treatment of melanoma, particularly with the introduction of targeted therapies and immunotherapies for patients with advanced melanoma (Garutti et al 2022). These treatments have been successful in the metastatic setting and have now progressed to the adjuvant setting, where they can benefit high-risk patients. High-risk melanoma is defined as a deep invasive primary tumor with or without ulceration (AJCC (8th edition) stage IIB and IIC) or with regional nodal disease (AJCC stage III). The 10-year melanoma-specific survival rates range from 84% for AJCC stage II down to 69% for AJCC stage III (Gershenwald et al 2017). While patients with thinner melanomas can be cured by surgery alone, increasing tumor thickness is associated with the risk of relapse and metastatic disease. Therefore, the healthcare provider and the patient must decide when to use adjuvant therapy, whether to treat in the adjuvant setting or wait until recurrence, and whether the benefits of adjuvant therapy outweigh the risks.

Adjuvant therapies such as immune checkpoint blockade or targeted therapy have been approved and are now considered standard of care not only for high-risk patients but also for intermediate-risk patients in AJCC stage IIB. These therapies have shown improvements in disease-free survival (DFS) and distant metastasis survival (DMFS), which can serve as a surrogate parameter for overall survival (Kobeissi and Tarhini 2022; Long et al. 2022). Adjuvant therapy is considered potentially curative and can prevent relapse and the poor outcomes seen in metastatic disease. In stage IV, adjuvant treatment with PD1-antibodies (ICI) ± CTLA4-antibodies (cICI), has also been demonstrated to be very effective and is, therefore, increasingly used in daily routine (Livingstone et al. 2022).

The toxicity of targeted therapy, dabrafenib and trametinib, is characterized by symptoms such as fever, gastrointestinal symptoms, joint pain, a decrease in the left ventricular function, and eye disorders (Lazaroff and Bolotin 2023) that can also impair quality of life (Scarpato et al. 2022; Lai-Kwon et al. 2023) In contrast, immune checkpoint inhibitors (c(ICI)) can induce autoimmune toxicity in nearly every organ system and a small subset of patients with a fatal course (Wang et al 2018). Despite the frequency of side effects, in the majority of patients, health-related quality of life (HrQoL) is not or only temporarily impaired (Bottomley et al. 2021; Khattak et al. 2022; Pedersen et al 2023; Lai-Kwon et al. 2023). However, in case of severe side effects, HRQoL may be persistently impaired, which can eventually lead to treatment stop (Pedersen et al. 2023; Wang et al. 2018).

In contrast to TT, (c)ICI has the potential for severe side effects that may be chronic and may be fatal or accompanied by a deterioration for quality of life (Wang et al. 2018; Schulz et al. 2022). Therefore, it is important to discuss the risks and benefits of therapy with the patient considering the benefits and risks of the treatment. Especially in adjuvant treatment, physicians need to discuss the individual risk–benefit ratio with eligible patients thoroughly. The physician’s beliefs may influence this decision-making process, so it is important to be aware of any differences between patients’ and physicians’ attitudes toward toxicity. Currently, there is limited knowledge about the differences between melanoma patients and their physicians concerning their preferences toward the toxicity of adjuvant melanoma treatment (Krammer et al. 2014; Weiss et al. 2020). However, it is known that patients and physicians may rate the benefit–risk ratios differently in other tumor entities (Zhang et al. 2023).

We conducted a study to evaluate the attitudes of dermatooncologists towards toxicity during adjuvant treatment and compared the results with a patient cohort we previously evaluated in these melanoma centers (Kähler et al. 2023). We aimed to identify any differences between melanoma patients and their treating physicians regarding the risk–benefit of adjuvant treatment in melanoma. There is limited data available about physician preferences for benefit versus toxicity in these treatments in the adjuvant setting. This study is the first to investigate, in a multicenter approach, how dermatooncologists value different spectrums of toxicity of adjuvant immunotherapy and targeted therapy in direct comparison to their patients.

## Methods

### Physicians, patients and study centers

This is a cross-sectional, observational, non-interventional questionnaire study that involved ten German and one Swiss skin cancer center with high expertise in treating melanoma. The study included dermatologists and dermatology residents who were familiar with the treatment of melanoma and worked in a German/Swiss melanoma center where melanoma patients are diagnosed, resected, and treated with systemic therapy by dermatooncologists.

The group of physicians was compared with previously evaluated patients with low-risk melanoma, defined as T1a, at least 8 weeks after initial diagnosis, no sentinel node biopsy or significant co-morbidities (Kähler et al. 2023). The rationale for low-risk melanoma patients was to choose a patient cohort with the experience of melanoma diagnosis, but not in the situation of having to decide for or against adjuvant treatment, to avoid ethical conflicts potentially induced by this study that may influence a patient’s decision.

We collected information on various sociodemographic factors, including age, gender, marital status, employment status, and working hours. We also asked about previous experience with cancer and co-morbidities. Additionally, we evaluated professional data such as the frequency of contact with melanoma patients, frequency of prescription of adjuvant treatment, duration of being a dermato-oncologist, percentage of subjects treated with mild side effects, and percentage of subjects treated with severe toxicity.

### Treatment trade-off

A survey tool that met the objective of our study was not available, so we created a new questionnaire. The questionnaire’s treatment scenarios were based on the literature and the expertise of two clinical dermato-oncologists. Pre-testing of the questionnaire for comprehensibility was done by three independent physicians and four volunteering patients provided feedback was used to revise it.

To elicit preferences, we used a paper-based treatment-trade-off task. Participants were asked to imagine having melanoma with a 30% chance of 5-year DFS and a 50% chance of 5-year OS. We described three treatments (TT, ICI, or cICI treatment), including the nature and probability of side effects, and asked participants to choose their preferred treatment. Additionally, we evaluated preferences for the recurrence of melanoma after adjuvant treatment, resulting in 12 different scenarios (an example is provided in the supplementary).

Scenario 1 = TT without side effects.

Scenario 2 = TT with mild to moderate side effects.

Scenario 3 = TT with severe side effects.

Scenario 4 = ICI without side effects.

Scenario 5 = ICI with mild to moderate side effects.

Scenario 6 = ICI with mild to moderate side effects and abnormal blood values.

Scenario 7 = ICI with severe side effects.

Scenario 8 = cICI without side effects.

Scenario 9 = cICI with mild to moderate side effects.

Scenario 10 = cICI with mild to moderate side effects and abnormal blood values.

Scenario 11 = cICI with severe side effects.

Scenario 12 = Recurrence of melanoma after adjuvant treatment (only rated for acceptability).

In contrast to previous uses of treatment-trade-off, participants were not presented a series of different DFS and OS rates for each scenario (1). Instead, the participants were requested to specify the minimum number of prevented recurrences or deaths necessary for them to opt for the treatment instead of not receiving any treatment. In other words, they were asked to state the required chances of DFS and OS that would make them choose the treatment over the alternative of no treatment. The statement to be completed, for example, “I would choose the treatment described in scenario 1 if it would prevent a relapse in at least ___ of these 70 patients.”

Participants were additionally asked to rate the acceptability of each scenario using visual analog scales (VAS) ranging from 0% = completely unbearable to 100% = completely bearable.

Thus, for each scenario, participants rated the minimally required increase in DFS and OS, respectively, as well as acceptability using the VAS.

### Primary endpoint

The primary objective was to determine preferences for adjuvant treatment with severe side effects in terms of the minimum required benefit, as defined in the treatment trade-off task, which was an additional chance of 5-year DFS.

### Secondary endpoints

To identify preferences for adjuvant treatments with mild to moderate and severe side effects during (c)ICI and TT, we needed to determine the minimum benefit required in terms of the additional chance of 5-year DFS and 5-year OS. This will be stated in the treatment trade-off task.

### Additional assessments

We asked physicians to rate their preference for infusion or oral medication on a 5-point scale from “completely agree” to “do not agree at all”.

*Self-applied medication*: “It is okay for me to take the medicine on my own”.

*Supervised medication*: “It seems beneficial to me to have the drug administered under the supervision of a doctor”.

*Rather visits than self-application*: “I’m happy to put up with infusions and more frequent visits to the doctor, as long as I then don’t have to be responsible for taking the medicine myself”.

*Acceptance of long doctor’s appointments*: “I can accept that an appointment with infusion and medical examination can take several hours”.

*Compliance with a strict intake schedule*: “I can stick to a precise schedule for taking pills”.

Importance of treatment method (infusion vs. pill): “The way I get the medicine administered (infusion or pills) matters to me”.

In addition, participants rated their preference for dosage via infusion vs. pill on a horizontal VAS from − 100 (infusion) to + 100 (pills) and 0 indicating “undecided”.

The same data as previously for patients (Kähler et al 2023) were assessed in this second part of the study for physicians: preferences, socio-demographics, and self-experience with cancer.

### Sample size calculation

The number of participants to be included was determined according to the primary endpoint of preferences for BRAF/MEKi treatment. To determine the percentage of participants who would choose BRAF/MEKi treatment at a 5-year-DFS of 65% or lower with a 95% confidence interval width of ± 10 percentage points, 104 analyzable data sets were needed (or less if the distribution of participants would differ from 50:50; calculated with PASS Sample Size 2008).

### Statistical approach

For all variables, descriptive statistics were computed (frequencies, percentages, mean, median, and/or standard deviation (SD), as applicable).

Participants were excluded from the OS, DFS, or VAS analysis, respectively, if they misordered two or more pairs of scenarios (e.g. lower rank for mild-to-moderate side effects than for severe side effects in otherwise identical scenarios) as this was regarded as an indicator of insufficient understanding of the rating task.

OS, DFS, and VAS were analyzed as the arithmetic mean along with the 95% confidence interval. Differences between treatment scenarios were tested with paired samples t tests. Preferences for the different scenarios (PFS, OS, VAS) were tested for differences for statistically significant differences between treatment scenarios and between physicians and patients using paired samples t tests.

Significance levels equal to or below 0.05 were considered statistically significant; no adjustment for multiple testing was performed.

The association of treatment preferences (DFS, OS, VAS) with important characteristics (socio-demographic data, self-experience with cancer, psychological constructs) was assessed using bivariate tests (Pearson correlations or t-tests, depending on variable scaling).

## Results

**Fig. 1:**
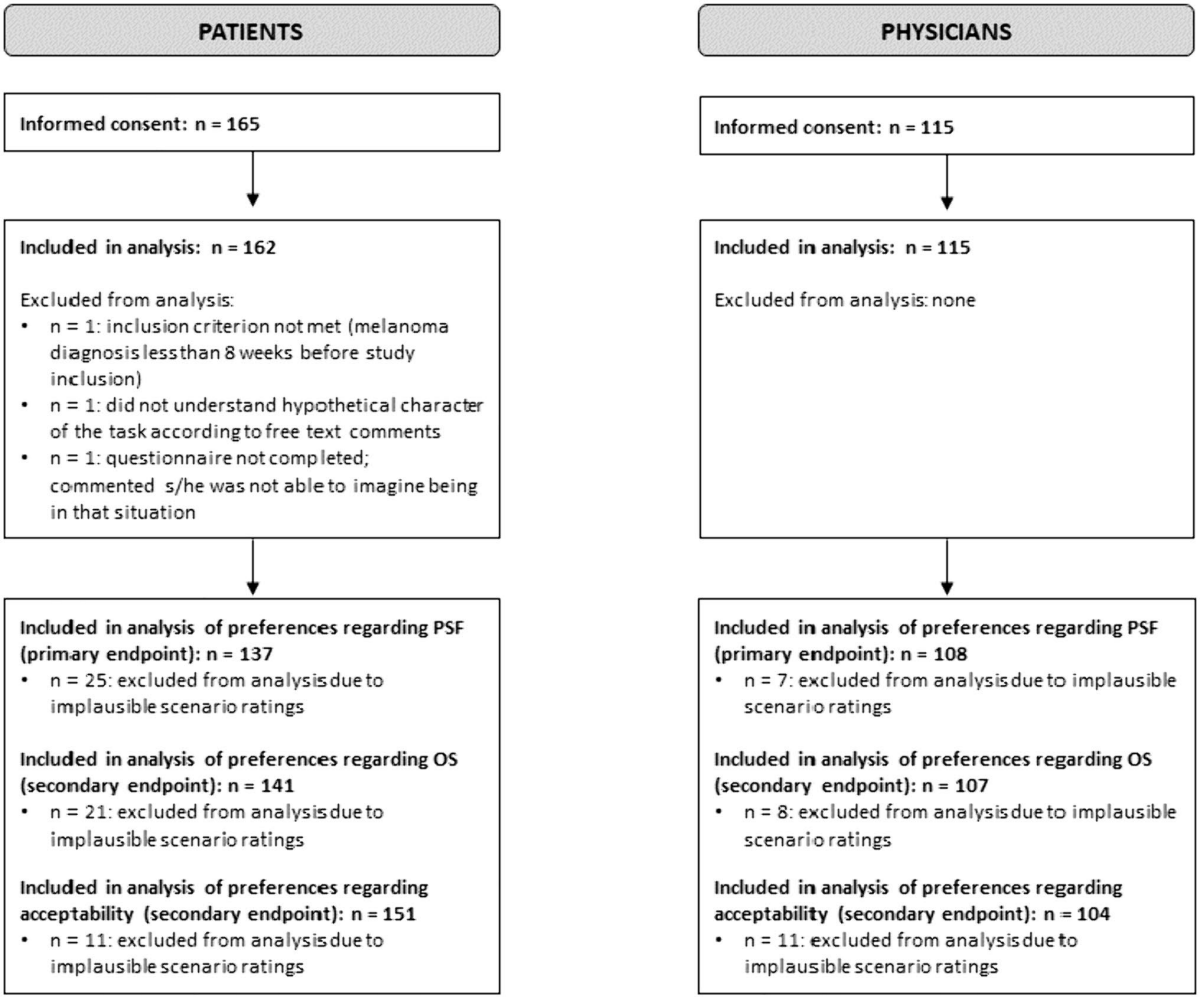
Study flowchart represented included, excluded, and analyzed physicians in comparison to previously analyzed patients (Kähler et al. 2023)

All 115 physicians who gave informed consent could be included in the analysis. From the analysis of the different scenario ratings, between 7 and 11 questionnaires had to be excluded, with n = 108 analyzable for the primary endpoint (Fig. 1). Out of 165 patients who have been analyzed in a previous part of the study (Kähler et al. 2023), 3 had to be excluded from analysis for different reasons. Regarding the analysis of the scenario ratings, between 11 and 25 patients had to be excluded, with n = 137 analyzable for the primary endpoint.

### Characteristics of physicians

Dermatologists were between 25 and 60 years of age (mean 33.9 years, median 32.0, SD 7.7), 67% were female. Most were in a relationship or married and living with one person. Median working hours were 42 per week (Table 1).

**Table 1:** Physician characteristics: sex, family, living, income, nationality, job (*n* = 115)

	Frequency	Percent
Sex		
Female	77	67.0
Male	37	32.2
Number missing	1	0.9
Family		
Single	22	19.1
Committed relationship	51	44.3
Married	38	33.0
Civil union	1	0.9
Number missing	3	2.6
Living		
Alone	28	24.3
With 1 person	56	48.7
With 2 persons	11	9.6
With 3 persons	11	9.6
With 4 persons	4	3.5
Not alone, but without number of persons stated	2	1.8
Number missing	3	2.6
Net income per month		
1600–below 2400 EUR	1	0.9
2400–below 4000 EUR	58	50.4
4000–below 4800 EUR	16	13.9
4800–below 5600 EUR	9	7.8
5600–below 6400 EUR	4	3.5
6400 EUR or more	7	6.1
Number missing	20	17.4

Physicians had a median of 40 patient contacts per month, according to Table 2. Their experience as a physician ranged from 0.1 to 35 years, with a median of 3.5. They reported that severe side effects were more common in patients during cICI (median 30%) than in TT or ICI (median 10%). The experience of physicians as clinical dermatologists or the frequency of consultations with melanoma patients did not correlate with scenario ratings, except for scenario 3 (severe side effects during TT), as shown in Table 3.

**Table 2:** Descriptive analysis: patient contacts, prescriptions and experience as a physician

	Valid	Missing	Mean	Median	SD	Minimum	Maximum
Melanoma patient contacts per month	101	3	80.4	40.0	105.9	0	800
Prescriptions of targeted therapies per month	99	5	6.3	2.0	8.8	0	50
Prescription of immune monotherapies per month	99	5	13.9	4.0	22.5	0	100
Prescription of combined immune therapies per month	99	5	4.5	1.5	6.4	0	40
Years as a clinical dermatologist	103	1	6.5	3.5	7.7	0.1	35
% of patients undergoing a targeted therapy with mild to moderate adverse effects	95	9	38.1	30.0	28.3	0	100
% of patients undergoing a targeted therapy with severe adverse effects	95	9	13.8	10.0	16.8	0	100
% of patients undergoing a monotherapy with mild to moderate adverse effects	95	9	36.0	30.0	26.1	0	90
% of patients undergoing a monotherapy with severe adverse effects	95	9	14.4	10.0	15.2	0	100
% of patients undergoing a combinated therapy with mild to moderate adverse effects	94	10	49.3	50.0	32.6	0	100
% of patients undergoing a combined therapy with severe adverse effects	94	10	29.7	30.0	21.3	0	100

**Table 3:**
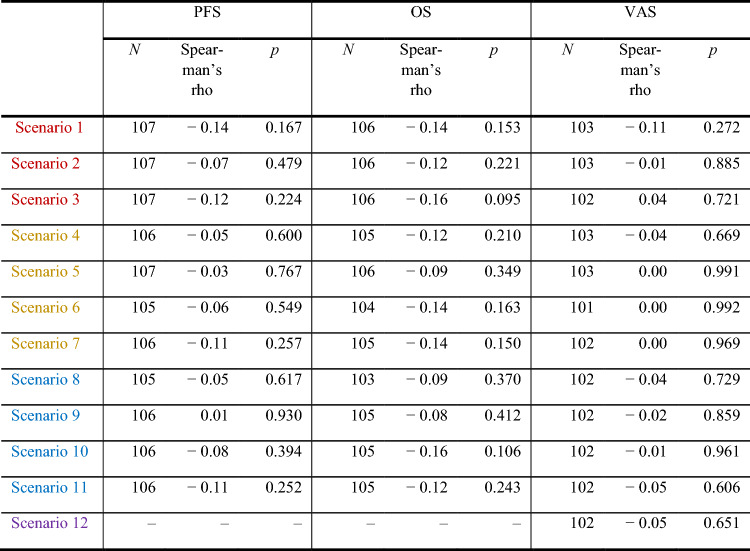
Correlation of years of experience working as a dermatologist with scenario ratings

### Patient characteristics

To describe the group of patients analyzed in our previous study, we present the socio-demographic characteristics of the full analysis set, which included 162 patients (Kähler et al. 2023). The patient cohort was predominantly German/Swiss (95%), with the remaining 5% having different nationalities. The group was almost equally divided between male (47%) and female (57%) subjects. The patients’ ages ranged from 24 to 93 years, with a median age of 60 years. The median time since melanoma diagnosis was 1 year (SD 5 years, range: 0–32 years). Most patients were married and living with one person, and the majority were either employed (with a median of 39 h/week) or retired.

### Scenario rating concerning disease-free survival

In various scenarios, physicians required significantly fewer prevented relapses to accept the treatment and its side effects compared to patients (Tables 4, 5, 6).

**Table 4:**
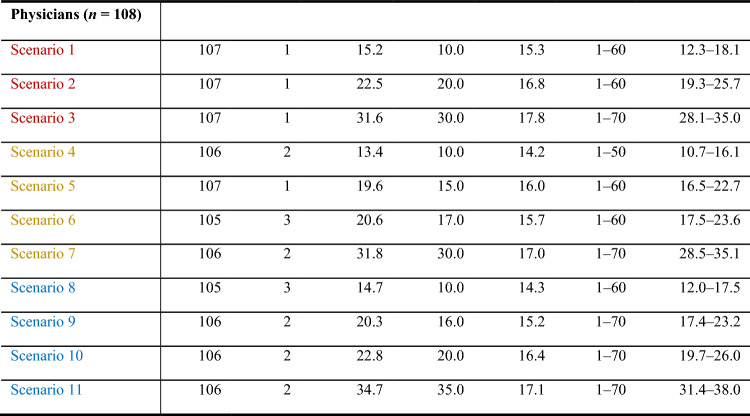
Scenario rating regarding DFS: minimal number of prevented relapses out of 70 that is needed for the treatment to be accepted (*n* = 108 physicians, red: TT, purple: ICI, blue: c(ICI))

**Table 5:**
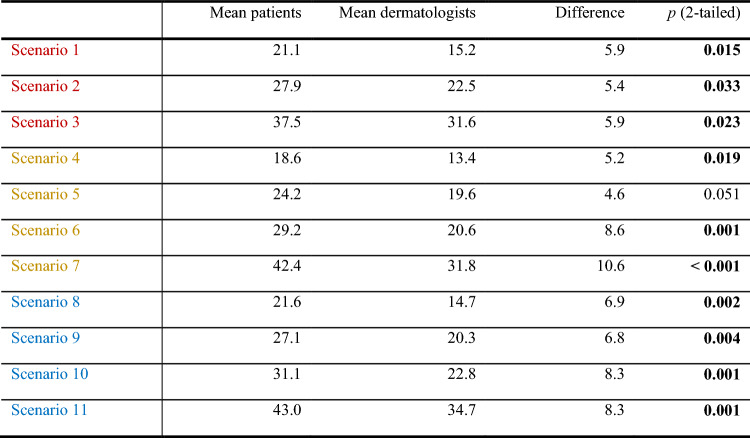
Differences between the scenario ratings by physicians and patients (Kähler et al. 2023) (red: TT, purple: ICI, blue: c(ICI))

**Table 6:**
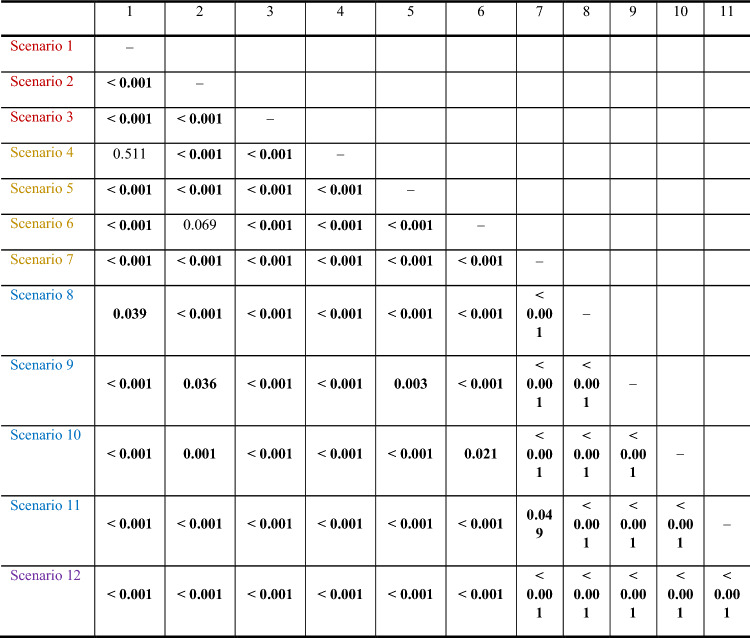
P-values of the paired t -tests on differences in mean scenario ratings regarding DFS in physicians (red: TT, purple: ICI, blue: c(ICI))

In case of severe side effects, physicians required for TT a median reduction of 30 out of 70 relapses (mean 31.6, SD 17.8, range 1–70, 95% confidence interval 28.1–35). Physicians needed identical numbers of relapses prevented for TT (30) and (c)ICI (35) compared to patients who required 15 additional prevented relapses at 5 yrs for (c)ICI (50) compared to TT (35). In other words, physicians required similar 5-year DFS rates for ICI and TT (60–65%). Patients needed a 15% improvement of 5-year DFS for ICI compared to TT (80%/65.0%).

In case of no side effects, both physicians and patients requested a similar reduction in relapses. The ratings were 10/10 for TT, 10/10 for ICI, and 10/15 for cICI. However, when mild-to-moderate side effects were present, the situation changed. Physicians requested a significantly lower number of prevented relapses compared to patients. The ratings were 20/30 for TT, 15/20 for ICI, and 16/29 for cICI. Most of the scenarios were statistically different from each other, as shown in Table 6.

### Scenario rating concerning overall survival

In case of no or mild-to-moderate side effects, physicians require a lower number of prevented deaths (5 and 10 for TT, 5 and 5 for ICI, 5 and 10 for cICI; median, Table 8) than patients (5 and 15 for TT, 5 and 15 for ICI, 10 and 20 for cICI; median, Kähler et al. 2023). Most physician ratings of scenarios were statistically different from each other (Table 7). Acceptance decreased with the severity of side effects. For TT with severe side effects, physicians and patients required a median of 15/25 avoided deaths and 20/30 avoided deaths for ICI or 20/35 deaths in case of cICI (*p* < 0.001), respectively.

**Table 7:**
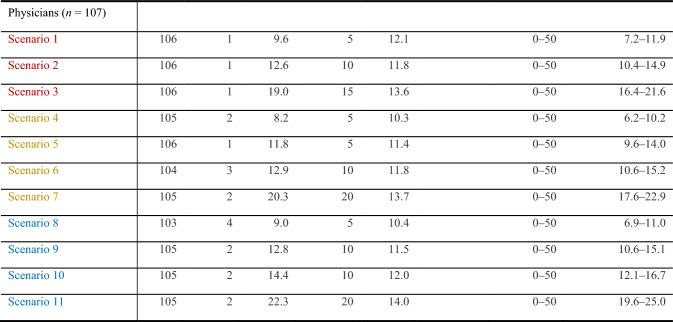
Scenario rating regarding OS: Minimal number of prevented deaths out of 50 that is needed for the treatment to be accepted (*n* = 107 physicians)

For survival, in case of mild-to-moderate side effects, physicians expected an equal OS improvement (+ 5 to 10%) for all three treatment modalities, whereas patients required an increase of 15–22% for 5-year melanoma survival (ICI + 18 to 22% compared to TT + 15%; Kähler et al. 2023). In case of severe toxicity, physicians expected an equal OS improvement (+ 15 to 20%) for all three treatment modalities, whereas patients required an increase of 25–35% for 5-year melanoma survival ((c)ICI + 30 to 35% compared to TT + 25%).

The average ratings regarding OS were statistically different for most scenarios in both patients and physicians (table not shown).

### Scenario rating by socioeconomic characteristics

There was a weak correlation (*r* < 0.3) between age and DFS ratings with older patients tending to require higher effectiveness to accept a treatment. In physicians, in contrast, gender, income, or co-morbidities did not show any association with DFS or OS rating.

### Impact of self-experience of cancer on scenario rating

3.5% of physicians experienced cancer themselves (Table 8). Additionally, 67% of physicians had close persons affected by cancer. Our study showed that previous cancer experience did not correlate with PFS, OS, and scenario ratings in either group, except for a significant OS rating for scenarios 8 and 9 (cICI without side effects or only with mild-to-moderate side effects) in the patient cohort who had previous cancer experience.

**Table 8:** Dermatooncologists’ self-experiences with cancer (*n* = 115)

	Frequency	Percent
Self-affected by cancer		
Yes, currently	0	0.0
Yes, in the past	3	2.6
Melanoma	1	0.9
Not specified	2	1.8
No	112	97.4
Number missing	1	0.9
Persons close to participant affected by cancer		
Yes, close relatives	67	58.3
Yes, partner	2	1.7
Yes, close friends	7	6.1
Yes, others	7	6.1
Ex-partner	1	0.9
Friends	1	0.9
Colleagues	1	0.9
Parents in law	2	1.7
Uncles/aunts	2	2.1
No	38	33.0
Number missing	1	0.9

The average ratings for PFS scenarios were similar for both patients with and without cancer experience. However, the average ratings for OS scenarios were also similar between the two groups, except for scenarios 8 and 9, which showed some differences (data not shown).

### Dosage form preferences: infusion vs. oral medication

90% of physicians and 63% of patients (Kähler et al. 2023) agreed that it was acceptable to take their medicine on their own (Table 9). Physicians were more likely to agree with this statement than patients. Both patients and physicians rather disagreed to prefer supervised medication, and there was no significant difference between the two groups in this regard.

**Table 9:** Absolute and total frequencies for medication preferences (*n* = 162)

	Patients (*n* = 162, Kähler et al. 2023)			Physicians (*n* = 115)			*p*
	Frequency	Percent	Cumulative %	Frequency	Percent	Cumulative %	
Self-applied medication							
Totally agree	100	63.3	63.3	102	89.5	89.5	–
Rather agree	25	15.8	79.1	9	7.9	97.4	–
Undecided	21	13.3	92.4	2	1.8	99.1	–
Rather disagree	12	7.6	100.0	0	0.0	0.0	–
Totally disagree	0	0.0	0.0	1	0.9	100.0	–
Missing	4	–	–	1	–	–	–
Supervised medication							
Totally agree	20	12.7	12.7	15	13.2	13.2	–
Rather agree	33	20.9	33.5	19	16.7	29.8	–
Undecided	33	20.9	54.4	23	20.2	50.0	–
Rather disagree	55	34.8	89.2	39	34.2	84.2	–
Totally disagree	17	10.8	100.0	18	15.8	100.0	–
Missing	–	–	–	1	–	–	–
Rather visits than self-application							
Totally agree	17	10.6	10.6	12	10.5	10.5	–
Rather agree	33	20.6	31.3	11	9.6	20.2	–
Undecided	27	16.9	48.1	12	10.5	30.7	–
Rather disagree	41	25.6	73.8	37	32.5	63.2	–
Totally disagree	42	26.3	100.0	42	36.8	100.0	–
Missing	2	–	–	1	–	–	–
Acceptance of long doctor’s appointments							
Totally agree	36	22.5	22.5	35	31.0	31.0	–
Rather agree	46	28.7	51.2	32	28.3	59.3	–
Undecided	29	18.1	69.4	17	15.0	74.3	–
Rather disagree	39	24.4	93.8	25	22.1	96.5	–
Totally disagree	10	6.3	100.0	4	3.5	100.0	–
Missing	2	–	–	2	–	–	–
Compliance to a strict intake schedule							
Totally agree	85	53.5	53.5	69	60.5	60.5	–
Rather agree	42	26.4	79.9	35	30.7	91.2	–
Undecided	21	13.2	93.1	4	3.5	94.7	–
Rather disagree	9	5.7	98.7	6	5.3	100.0	–
Totally disagree	2	1.3	100.0	0	0.0	0.0	–
Missing	3	–	–	1	–	–	–
Importance of treatment method (infusion vs. pill)							
Totally agree	64	40.3	40.3	29	25.4	25.4	–
Rather agree	38	23.9	64.2	33	28.9	54.4	–
Undecided	39	24.5	88.7	22	19.3	73.7	–
Rather disagree	15	9.4	98.1	16	14.0	87.7	–
Totally disagree	3	1.9	100.0	14	12.3	100.0	-
Missing	3	–	–	1	–	–	–
Do you currently use medication on a regular basis?							** < 0.001**
No	61	37.9	37.9	77	67.5	67.5	
Yes	100	62.1	100.0	37	32.5	100.0	
Missing	1	–	–	1	–	–	
Is there somebody who can accompany you to the infusions?							**0.041**
No	29	18.6	18.6	33	29.2	29.2	
Yes	127	81.4	100.0	80	70.8	100.0	
Missing	6	–	–	2	–	–	

On average, patients and physicians disagreed that they would accept infusions and doctor visits, with physicians being more opposed to this idea. However, both groups were willing to accept appointments that take several hours, and most of them stated that they could stick to a precise intake schedule, with physicians being more confident in this regard.

Interestingly, more patients than physicians had a strong preference for a particular administration method (infusion or pills), and this difference was highly significant (Table 10).

**Table 10:** Differences between the ratings by patients vs. physicians (1 = totally agree to 5 = totally disagree)

	Valid	Missing	Mean	Median	SD	Mini-mum	Maxi-mum	*p*
Self-applied medication								** < 0.001**
Patients	158	4	1.7	1.0	1.0	1	4	
Dermatologists	114	1	1.2	1.0	0.5	1	5	
Supervised medication								0.411
Patients	158	4	3.1	3.0	1.2	1	5	
Dermatologists	114	1	3.2	3.5	1.3	1	5	
Rather visits than self-application								**0.017**
Patients	160	2	3.4	4.0	1.3	1	5	
Dermatologists	114	1	3.8	4.0	1.3	1	5	
Acceptance of long doctor’s appointments								0.114
Patients	160	2	2.6	2.0	1.2	1	5	
Dermatologists	113	2	2.4	2.0	1.2	1	5	
Compliance to a strict intake schedule								**0.049**
Patients	159	3	1.8	1.0	1.0	1	5	
Dermatologists	114	1	1.5	1.0	0.8	1	4	
Importance of treatment method (infusion vs. pill)								**0.001**
Patients	159	3	2.1	2.0	1.1	1	5	
Physicians	114	1	2.6	2.0	1.3	1	5	

According to the results of the horizontal VAS, most physicians and patients preferred pills over infusions, with a median score of 31 and 41.5 (Table 11) on a scale ranging from − 100 (infusion preferred) to + 100 (pills preferred). However, a significant number of participants chose “0,” indicating no preference. The mean score was 26.1, with a standard deviation of 61.6 and a range of − 100 to 100, based on a sample size of 161 patients. Patients generally did not see any benefits in supervised medication. On average, they disagreed with the idea of preferring infusions and doctor visits, and tended to prefer appointments that took several hours. Patients also stated that they could adhere to a precise intake schedule and that the method of administration (infusion or pills) was important to them.

**Table 11:** Medication preferences (− 100 = preference for infusion; 100 = preference for pills)

	Valid	Missing	Mean	Median	SD	Minimum	Maximum	*p*
Infusion vs. pill								0.229
Patients	161	1	26.1	31.0	61.6	− 100	100	
Patients	114	1	34.6	41.5	53.9	− 100	100	

## Discussion

Our study revealed that patients and physicians have differing perspectives on toxicity during adjuvant therapy. The doctor´s view has been evaluated in dermatooncologists of 11 melanoma centers.

### Association with physicians’ and patients’ characteristics

Noteworthy, the group of physicians is healthier, younger, more educated, wealthier, and less affected by previous cancer diagnoses compared to the patient cohort (Kähler et al. 2023).

In the physician cohort factors such as age, gender, professional experience, and intensity of contact with melanoma patients did not show any correlation with the ratings of scenarios related to melanoma treatment.

For patients, we know that their ability to communicate treatment side-effects, comorbidities, and their view on the treatment risk/benefit profile has been identified as a critical driver of clinical decisions in adjuvant AJCC stage III disease (Livingstone et al. 2021).

Older melanoma patients tended to require higher effectiveness to accept an adjuvant treatment, this was reflected in the DFS ratings (Kähler et al 2023). The effect sizes were small though. These results are similar to our previous GERMELATOX analysis that evaluated patient preferences for adjuvant interferon-alpha (Kähler et al. 2016). In contrast, Weilandt et al. showed in a discrete choice approach in melanoma patients that increasing age, toxicity, and impact on their daily routine were more relevant than efficacy (Weilandt et al. 2021). They also found that married patients and patients with a higher level of education have higher expectations of treatment efficacy (Weilandt et al. 2021). In our study, a pre-existing cancer diagnosis did not influence average scenario ratings regarding acceptability or DFS in patients (Kähler et al 2023). Average scenario ratings regarding OS also did not differ between patients with experience with cancer and those without, except for scenarios 8 and 9 (cICI without or only mild to moderate side effects) but, again, with small effect sizes only (Kähler et al. 2023).

Physicians should be aware of the difference in perspectives between the patient and themselves and guide the informed consent process accordingly. Atkinson et al. found that physicians favored adjuvant therapy for their patients in 35% of cases, favored observation in 35% of cases, and had no preference in 29% of cases. Although these preferences were not communicated to the patients, the patient´s choice regarding adjuvant therapy (treatment vs. observation only) showed a significant, albeit small, correlation with the physician’s preference (treatment vs. observation only/no preference) (Atkinson et al. 2023).

### Difference between the perception of TT versus (c)ICI

In our study, physicians and patients had different perspectives on the side effects of TT and ICI. Physicians were less concerned by IO toxicity and potentially long-lasting side effects and generally had a less negative view of toxicity maybe due to their awareness of the benefits of successful side effect management. Patients, on the other hand, were more willing to accept severe side effects induced by TT compared to (c)ICI. Patients rated potentially lethal or not resolving side effects induced by (c)ICI worse. However, most of the scenarios were rated as completely unacceptable by less than 1% of the patients and 0% of the physicians, showing the immense willingness of German and Swiss patients to tolerate treatment-related side effects.

Interestingly, patients were more willing to accept TT-associated pyrexia if the drug efficacy and, therefore, their outcome benefit is known (Mansfield et al. 2021), physicians should focus on precise and adequate information in the informed consent process.

The more negative perception of severe side effects during adjuvant treatment with (c)ICI compared to TT has also been confirmed by the comparison of the acceptability of scenarios. This can be explained by the possibility of long-lasting toxicity with sequelae and as well potentially fatal course of autoimmune side effects. A trial with structured interviews of melanoma physicians and nurses identified severe immune-related treatment side-effects overall and recurrence-free survival as highly influential factors in their immunotherapy decision-making (Livingstone et al. 2020). Melanoma patients scored higher on HRQoL social well-being at pre-treatment of ICI, were more likely to endorse positive statements about adjuvant immunotherapy, and perceived that their physician preferred adjuvant therapy combined with lower decisional regret and higher satisfaction, even if they experienced toxicity or recurrence (Atkinson et al. 2023). This may have an impact on patient–physician discussions and patient reflection at the time of treatment choice. Decisional regret was also lower in patients in that trial who had undergone lymph node dissection consistent with the idea that more aggressive treatment may be associated with less decisional regret (Atkinson et al. 2023). Therefore, the aspect of decisional regret should be considered and communicated in informed consent processes.

The difference in the mode of administration between c(ICI) and TT might also be a reason for melanoma patients to rate TT superior to (c)ICI. Most patients and dermatologists in our trial stated it was acceptable for them to take the medicine on their own. For dermatologists, this was significantly more the case than for patients. The majority did not see benefits in supervised medication. Most patients and dermatologists stated they could stick to a precise intake schedule, which was even more pronounced in dermatologists. More patients than dermatologists stated having a strong preference for an administration method (infusion or pills), which was highly significant. Our patients preferred the autonomy of an oral medication whereas the melanoma cohort of Weilandt and co-workers showed in their analysis that patients favored infusions. This might be explained by the fact that in our patient cohort, the decision for melanoma treatment and treatment regimen was an entirely fictitious scenario. Therefore, our patients might value the autonomy of an oral medication higher, whereas patients facing the adjuvant treatment decision in a real scenario perhaps might somewhat be overwhelmed by the challenge of understanding the process and therefore prefer to delegate the treatment responsibility regarding medication intake to their physician. Stellato and co-workers showed in a Canadian cohort that physicians assigned the highest preference for orally administered treatments (corresponding to the dosing regimen for dabrafenib–trametinib), melanoma patients had a similar preference for orally administered treatments and infusions administered over 30 min every 3 weeks (corresponding to the dosing regimen for pembrolizumab) (Stellato et al. 2019).

### Do current treatment options meet our expectations?

We observed that physicians tend to accept a less significant treatment benefit by adjuvant treatment as compared to patients. This might be because physicians are not personally involved in the treatment process. A study by Weiss et al. suggests that previous cancer experience could affect treatment outcome ratings. Patients and physicians who have had personal cancer experience tended to value life prolongation by melanoma treatment more positively than healthy controls or physicians without personal cancer experience (Weiss et al. 2020).

In our trial, physicians tended to rate melanoma relapse less negatively than patients (Kähler et al. 2023), which could be due to their more optimistic view of treatment options and outcomes.

For DFS patients’ expectations towards efficacy differed between the three treatment modalities only by a range of 6 percentage points, despite the distinct rate of grade 3–4 adverse events (ranging from 14.4 to 71.0%, Table 12, Kähler et al. 2023).

**Table 12:** Comparison between physician (ph) and patient preferences (pa) and efficacy demonstrated in clinical trials: DFS (Kähler et al. 2023)

5y-DFS (%)	Expected efficacy (all grades of tolerability) ph vs. pa	Expected efficacy (in case of severe side effects) ph vs. pa	Grade 3–4 side effects (%) in clinical trials	Efficacy in clinical trials
TT	50 vs. 55	60 vs. 65	41	52 (Dummer et al.)
ICI	48 vs. 57	60 vs. 68	14.4	47.1–66.4 (Larkin et al.)
cICI	50 vs. 62	65 vs. 71	71	64.2 (4y, Livingstone et al.)

In general, the clinical trials conducted by Dummer et al. (2020), Larkin et al. (2023), Livingstone et al. (2022), and Schadendorf et al. (2022) have shown that the treatment efficacy is capable of meeting the expectations in terms of DFS found in our physician cohort. However, some follow-up data are still immature, and for cICI, only 4-year DFS data are available so far. Physicians and patients expected higher DFS rates for TT and ICI in case of severe side effects than shown for these treatment options in clinical studies (Kähler et al. 2023). In this situation, the efficacy would not be high enough for the patients. For OS (Table 13), the results are similar, but in case of severe side effects, the gap between expectations and the efficacy demonstrated in clinical trials so far seems to be smaller in case of using cICI. The gap between the expectations from risks and benefits of TT and ICI is more noticeable compared to cICI. This suggests that patients may not value the risk–benefit ratio as much as they do for cICI. However, it is important to note that cICI is only administered to specific patients in the adjuvant setting in AJCC stage IV.

**Table 13:** Comparison between physician (ph) and patient preferences (pa) and efficacy demonstrated in clinical trials: OS (Kähler et al. 2023)

5y-OS (%)	Expected efficacy (all grades of tolerability) ph vs. pa	Expected efficacy (in case of severe side effects) ph vs. pa	Grade 3–4 side effects in clinical trials	Efficacy in clinical trials
TT	60 vs. 65	65 vs. 75	41	64.6–75.3 (5y DMFS, Schadendorf et al.)
ICI	60 vs. 68	70 vs. 80	14.4	58 (5y DMFS, Larkin et al.)
cICI	62 vs. 71	70 vs. 85	71	83.3 (4y OS, Livingstone et al.)

In contrast, the expectations of the physician cohort for OS are concordant with the results from clinical trials for the available treatment options, as well as for severe toxicity.

Krammer et al. showed in their trial that attitudes towards toxicity and benefit vastly differed between healthy participants, physicians and melanoma patients. Whereas melanoma patients showed a high willingness to endure side effects despite very small survival gains (down to 1 extra week) or even only hope with no survival benefit, healthy controls were more critical, while physicians were the most therapy adverse (Krammer et al. 2014). Stellato et al. described that patients preferred an increased probability of remaining cancer-free over 21 months whereas physicians prioritized remaining alive over 36 months (Stellato et al. 2019).

### Limitations of the study

Our study had some limitations. Firstly, the physician cohort we selected was mostly female and younger than a typical patient cohort; however, this corresponds with the typical composition of this group of persons. Secondly, we used a previously analyzed patient population with only low-risk melanoma as surrogates for those in later disease stages, due to ethical reasons. Thirdly, we did not analyze the perceptions of adjuvant melanoma treatment over time, so we may have missed possible changes in the individual course of the disease. However, evidence suggests that the tumor stage does not necessarily influence patients’ preferences (Atkinson et al. 2023). Fourthly, the usual melanoma patient cohort consists of more male than female patients, while in our study, more female patients were willing to participate. Finally, patient preferences were elicited based on hypothetical scenarios, which may not be completely comparable to real-life treatment decisions.

Overall, our study revealed a significant information and knowledge gap between physicians and patients, indicating different perspectives on treatment side effect perception. The most important goal should be to increase patients’ confidence in current treatment modalities and the competence of their physician. Physicians should be able to change their perspective to improve their understanding of possible reasons for patients declining adjuvant treatment.
